# Data Science and Predictive Analytics: Biomedical and Health Applications Using R

**DOI:** 10.5195/jmla.2020.901

**Published:** 2020-04-01

**Authors:** Benjamin H. Saracco

**Affiliations:** Library, Cooper Medical School of Rowan University, Camden, NJ, saracco@rowan.edu, http://orcid.org/0000-0002-6049-0262

Ivo D. Dinov’s *Data Science and Predictive Analytics: Biomedical and Health Applications Using R* is a comprehensive twenty-three-chapter text and online course for burgeoning or seasoned biomedical and/or health sciences professionals who analyze data sets using the R programming language. Accompanying the book is a freely available, Creative Commons Attribution-ShareAlike (CC-BY-SA) and Lesser General Public License (LGPL) massive online open course (MOOC) with even more instructional technology tools to assist learners, such as class notes, video lectures by the author, example data sets, and sample R libraries that can be used in the book and course’s assignments. The text is available in hardcover, softcover, or electronic book format.

While other recent texts have been published focusing on the topic of using R to analyze health and biomedical statistics, those resources were tailored to specific health professions, such as epidemiologists, or specific statistical analysis software, such as SAS [1, 2]. Dinov’s *Data Science and Predictive Analytics* is broader in scope and coverage and provides instructional information for any student or professional working with “Big Data” in the health or biomedical science fields. Dinov explains upfront that his text and accompanying MOOC are designed for learners at different skill levels. The chapters in *Data Science and Predictive Analytics* can be completed sequentially or individually, depending on the learner’s interest or prior experience working with the R programming language. The MOOC provides learners with a self-assessment pretest on basic mathematical and statistical knowledge that the learner needs to have to successfully complete the exercises that are featured in *Data Science and Predictive Analytics*. The author shrewdly supplies links to freely available resources that can be used for remediation, if needed.

*Data Science and Predictive Analytics* may seem intimidating in length and scope to early year graduate students or early career professionals who are still developing their data science and/or predictive analytic skills, but beginning learners will soon find that this resource is designed to be digested as a self-paced guide, where learners can choose their own learning paths. The MOOC provides learners a dynamic flowchart for those who are interested in mastering only specific topics covered in *Data Science and Predictive Analytics*, à la carte style. This provides thoughtful flexibility and choice for learners with varying informational needs and educational backgrounds.

In the field of medical and health sciences librarianship, this text and its MOOC would be an excellent purchase for anyone beginning to provide introductory or advanced data services to their patrons. Additionally, *Data Science and Predictive Analytics* is useful for library professionals who are interested in developing instruction on topics ranging from the foundations of the R programming language covered in chapter two, data visualization in chapter four, or natural language and text mining in chapter twenty.

*Data Science and Predictive Analytics* provides learners a comprehensive text and accompanying MOOC for any professional who is interested in augmenting their data sciences skill sets. It is a resource that would be a useful addition to academic, health sciences, or medical library collections.

***Benjamin H. Saracco, MLS, MAIT***, saracco@rowan.edu, http://orcid.org/0000-0002-6049-0262, Library, Cooper Medical School of Rowan University, Camden, NJ
